# The implementation effect of DIP payment method across different population in Southwest China based on multi-group interrupt time series

**DOI:** 10.3389/fpubh.2025.1572475

**Published:** 2025-06-02

**Authors:** Lixiang Wu, Ni Wu, Yuhan Cao, Xiaoyuan Zhou

**Affiliations:** ^1^West China School of Public Health, West China Fourth Hospital, Sichuan University, Chengdu, China; ^2^School of Public Health, Chengdu University of Traditional Chinese Medicine, Chengdu, China

**Keywords:** Diagnosis-Intervention Packet, mandatory medical insurance, medical services quality, the burden of patients, medical efficiency

## Abstract

**Background:**

Since 2020, China has implemented a payment method known as “Diagnosis-Intervention Package” (DIP) in 71 cities nationwide to address the specific needs of the country. The objective of this study is to evaluate the impact of DIP on medical quality and the burden experienced by inpatients covered under the Urban Employee Basic Medical Insurance (UEBMI) and Urban and Rural Residents Basic Medical Insurance (URRBMI). Furthermore, it aims to investigate potential differences in these effects between inpatients enrolled in the two distinct types of insurance, thereby enhancing our understanding of how this reform in payment methods influences healthcare delivery, and refine the social security system.

**Methods:**

We conducted a multiple-group interrupted time series analyses (MGITSA) on outcome variables reflecting medical services quality, and the burden of UEBMI and URRBMI inpatients, based on a dataset containing 180,071 inpatient reimbursement records in City C spanning from January, 2019 to December, 2021. This dataset included 42,581 records for URRBMI inpatients and 137,490 records for UEBMI inpatients.

**Results:**

After DIP implementation, both UEBMI and URRBMI showed increased inpatient numbers (21.59% and 22.26%, respectively), reduced LOS (7.10% for UEBMI, 0.29% for URRBMI), and higher ACR (3.07% for UEBMI, 15.36% for URRBMI). Hospitalization costs increased slightly for both groups (2.97% for UEBMI, 10.44% for URRBMI). Subgroup analysis revealed age-specific differences: significant LOS and cost changes in <18-year-olds and >45-year-olds, but minimal effects in 18–45-year-olds. MGITSA showed URRBMI experienced significant LOS reduction (β3=−0.004, P=0.014), while UEBMI had more pronounced LOS and ACR trends, with no significant inter-group differences in cost slopes.

**Conclusion:**

DIP improved hospital efficiency (reduced LOS, increased admissions) and financial protection (higher ACR) for both insurance groups in the short term, though hospitalization costs rose, requiring attention to potential service intensity inflation or cost-shifting. Age disparities in DIP impacts highlight the need for targeted policies. Continuous monitoring and policy adjustments are essential to balance cost control, service quality, and equity, ensuring DIP’s long-term effectiveness in China’s healthcare reform.

## Introduction

Global spending on health has been rising over the past decades, peaking at US$ 9.8 trillion in 2021 (1). Hospital service, has consistently accounted for one of the biggest shares in total healthcare expenditures in all countries across the world (2). In China, the healthcare cost has soared, especially in the past two decades, due to an aging population and the increasing prevalence of chronic diseases, which has put great pressure on the mandatory healthcare insurance systems (3, 4). To address this issue and improve hospital care quality, the Diagnosis-Intervention Packet (DIP) payment method was introduced in China.

DIP, as a specific payment method of China’s social security system in the medical field, was designed to categorize acute inpatient cases based on integrated diagnoses and procedures. It directly utilizes the International Classification of Diseases (ICDs) for its diagnostic coding system, enabling seamless integration with existing hospital health information systems. Due to the distinct grouping methodologies, CHS-DGR encompasses over 600 diagnosis-related groups currently in China, while DIP consists of 9,520 groups for diagnosis/procedure (5). Compared with DRG in Germany, which realizes cost control through standardized case grouping, DIP in China has formed a more detailed disease classification system based on historical data mining (6). Different from the fixed rate model of medical insurance in the United States, DIP innovatively incorporates the dynamic adjustment factors of regional economic differences (7). It is worth noting that China’s DIP payment scheme has added medical quality monitoring indicators to the payment rules. This design paradigm, which combines cost control and service quality, reflects the unique consideration of balancing the large-scale medical insurance system and regional resource differences.

Since 2019, China has encouraged regions to select either Diagnosis-Related Groups (DRG) or DIP as the principal payment method for their insurance risk pool, considering the uneven development of health information platforms across regions. By 2021, DIP had been used for actual payment in 71 pilot cities in China, and some non-pilot cities also customized the DIP grouping system according to local health insurance database (8). In City C, located in Southwest China, a significant adjustment to the DIP payment method was implemented on July 1st, 2020. Before this adjustment, the fee-for-service payment method was mainly used. This adjustment of DIP is the focus of our study. The adjusted DIP in City C consisted of approximately 1,200 groups to cover the costs of inpatient care at local hospitals. This adjustment aimed to further optimize the payment mechanism, improve the efficiency of medical resource utilization, and better control healthcare costs.

Previous studies on DIP have produced inconsistent results. Xie et al. (9) found that DIP could save hospital budget and lead to a notable improvement in hospital care efficiency. Lai et al. (10) believed that the DIP payment reform has achieved short-term success in slowing down the growth of medical expenses, while Qian et al. (11) reported that it increased inpatient medical costs. Moreover, most of the existing research has not focused on the differences in the impact of DIP on different types of medical insurance, such as UEBMI and URRBMI. The UEBMI covers employed adults and URRBMI provides reimbursement for non-formally employed individuals of all age – groups. Due to differing demographic profiles and premium collection mechanisms, financial protection varies across regions. In most areas, both insurance pools adopt the same payment method, that is, they make payments through DIP. Policymakers worry that payment method reforms might simultaneously and cohesively affect the health-related activities of both insured groups. Such effects could widen the gap in health service utilization and deepen economic inequalities among different population groups. Thus, understanding how DIP affects these two groups differently is crucial for policymakers. This knowledge can help ensure that the payment reform promotes fair access to healthcare services and does not widen the disparities in health service utilization between different population groups.

The objective of this study is to evaluate the impact of the adjusted DIP payment method on medical quality and the burden experienced by inpatients covered under UEBMI and URRBMI in City C. We also aim to investigate potential differences in these effects between the two types of insured inpatients, which will contribute to a more comprehensive understanding of the DIP payment reform and its implications for healthcare delivery in China and potentially offer valuable lessons for other countries facing similar healthcare payment challenges.

## Methods

### Study area and population

City C, situated in Southwest China, is one of the largest and most densely populated cities in the country. It comprises 19 municipal districts and 2 economic functional zones, with a population exceeding 15 million and a Gross Domestic Product (GDP) per capita of 94,622 *yuan* (approximately US$14,667). In terms of social security coverage, both UEBMI and URRBMI have nearly equal numbers of residents, with minimal imbalance, making it an ideal area to study the impact of DIP on different types of medical insurance.

### Study design

Since July, 1st, 2020, the adjusted-DIP payment method was implemented in City C, replacing the previous fee-for-service payment. We adopted an interrupted time series (ITS) design to assess the impact of this reform on inpatient services. The study period was from January 1st, 2019, to December 31st, 2021, with July 1st, 2020, as the intervention point. The pre-intervention stage was from January 2019 to June 2020, and the post-intervention stage was from July 2020 to December 2021. We compared the changes in inpatient services before and after the DIP implementation and evaluated the differences in effects between UEBMI and URRBMI inpatients at the same time period. By using a multiple-group interrupted time series analysis (MGITSA), we could make causal effect inferences to interpret the outcomes.

### Data source

We retrospectively collected medical claim data of inpatient care from four hospitals (including 2 tertiary general hospitals and 2 tertiary traditional Chinese medicine hospitals) in City C between January 1st, 2019 and December 31st, 2021. The data were obtained from the Hospital Information System (HIS), the frontpage of Medical Records Management System and Medical Insurance reimbursement System of the four hospitals. All data were anonymized to protect the privacy of individuals, and did not contain any personal information that could be traced back to a specific person. This dataset included patients’ demographic indicators, length of stay, hospital discharge status, and hospitalization costs, etc. After data screening, 180,071 cases were eligible for our study.

### Variables

To accurately quantify the trends of quality and economic burden of inpatient care in hospitals, we selected several variables based on existing literature and data availability (12). Medical efficiency variables, such as the numbers of inpatients, average length of stay (LOS) and average hospitalization costs per case, were used to evaluate the effectiveness of hospital care for different medical insurance inpatients. The economic burden of inpatients was represented by the actual compensation ratio per case (ACR) for different medical insurances (Equation 1).


(1) $$\mathrm{ACR}=\frac{\text{amount of medical insuranc}e\;\text{reimbursement}\;\mathrm{per}\;\text{case}}{\text{total hospitalization costs}\;\mathrm{per}\;\text{inpatient}\;}$$

All data in this study were adjusted using the relevant consumer price index (CPI), with 2019 serving as the base year (12). Skewed data distribution was addressed through log transformation.

### Descriptive analysis

Demographic characteristics of studied inpatients were described. In addition, an analysis was made on the changes of number of inpatients, average LOS, average hospitalization costs per case, and ACR for different types of medical insurance before and after the DIP implementation.

### Distribution of types of diseases

Since the quality and efficiency of medical services may be influenced by different types of diseases (13), we encoded each case with ICD-10 and used the initial letter of the ICD-10 code to classify discharged patients. Chi-square tests were conducted to explore potential differences in the proportion of diseases within each medical insurance system.

### Multiple-group interrupted time series analysis (MGITSA)

The MGITSA was utilized to compare the differences across patient groups within different types of medical insurance. Compared to a single group of ITSA, a multiple-group ITSA may be particularly valuable when there is an exogenous policy shift that affects all the groups (14).

In this study, patients covered by UEBMI were considered as the treatment group, and those by URRBMI were the control group, assuming that the two groups were affected by the same confounding factors.

We used a generalized linear segmented regression model followed by a Newey-West test to evaluate the impacts of the DIP payment reform on inpatient care in hospitals (15). According to the patients’ discharge date, Stata 16.0 was used to mark observations month by month (36 months in total) and perform all statistical analyses. The segmented regression model (Equation 2) is shown as below:


(2) $$\begin{array}{l} Y_{t}=\beta_{0}+\beta_{1}T_{t}+\beta_{2}X_{t}+\beta_{3}X_{t}T_{t}+\beta_{4}Z+\\ \beta_{5}{\mathrm{ZT}}_{t}+\beta_{6}{\mathrm{ZX}}_{t}+\beta_{7}{\mathrm{ZX}}_{t}T_{t}+\varepsilon_{t} \end{array}$$

**Figure 1:**
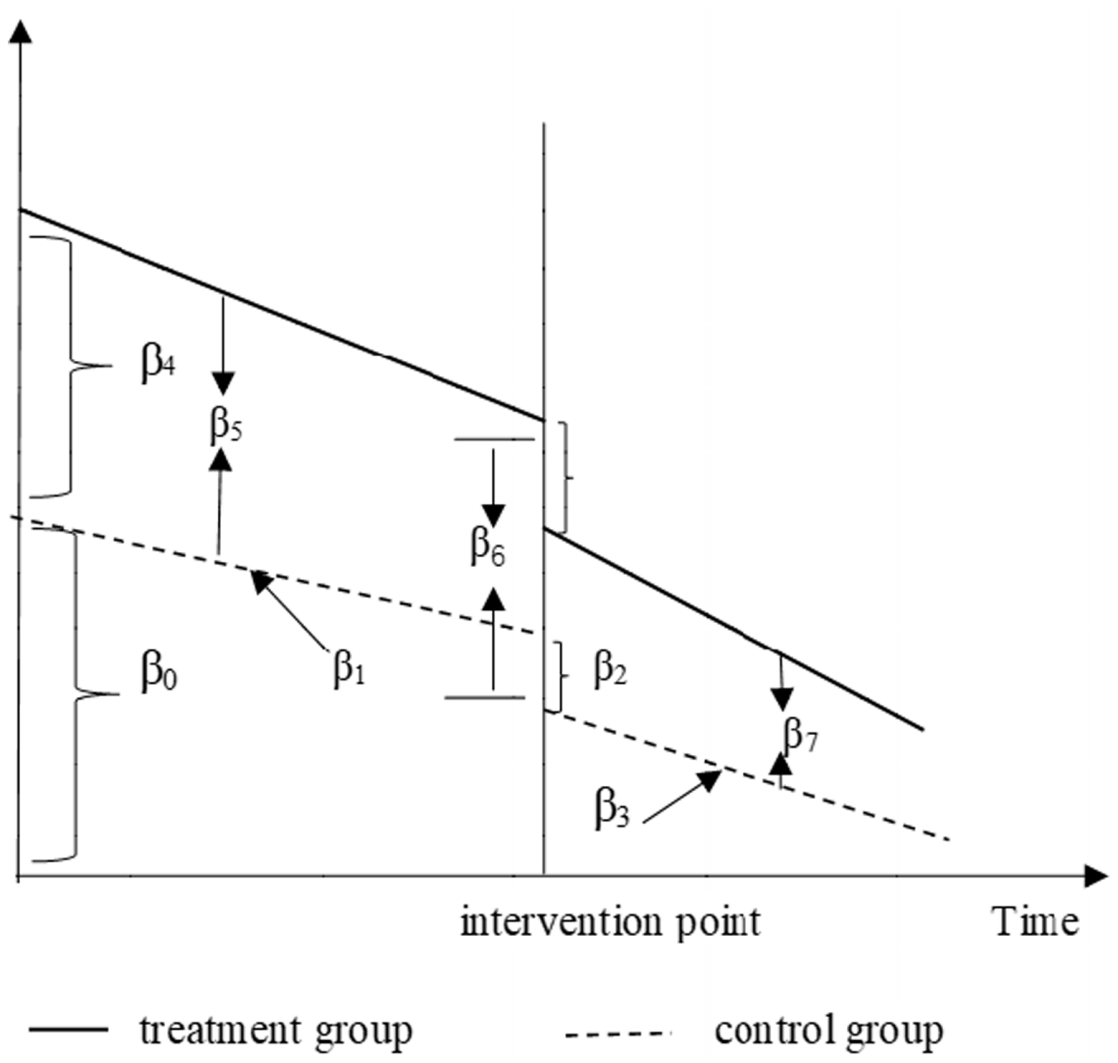
Visual depiction of two-group interrupted time-series analysis.

Here $Y_{t}$ is the outcome variable measured at each monthly point *t*. $T_{t}\;$ is the time series variable representing the time in months since the start of observation until time *t*, $X_{t}$ is a dummy variable representing the intervention (0 for the pre-intervention period and 1 for the post-intervention period), and *Z* is a dummy variable denoting the employees’ group for patients covered by UEBMI and residents’ group for those of URRBMI. $X_{t}T_{t}$ is an interaction term of the time and intervention, and ${\mathrm{ZT}}_{t}$, ${\mathrm{ZX}}_{t}$, and ${\mathrm{ZX}}_{t}T_{t}$ are all interaction terms among previously described variables. β_0_ is a constant term representing the initial value of the outcome variable. β_1_ represents the slope of the employees’ group before intervention, β_2_ defines the transient change of residents’ group at the time of intervention, β_3_ represents the difference in slope before and after the intervention in the residents’ group, and β_1_ + β_3_ represents the trend after the intervention (16). Meanwhile, β_4_ represents the difference in the level (intercept) of the outcome variable between the two groups prior to the intervention, β_5_ represents the difference in the slope (trend) of the outcome variable between the two groups prior to the intervention, β_6_ indicates the difference between the two groups in the level of the outcome variable at the time of intervention, and β_7_ represents the difference between the two groups in the slope (trend) of the outcome variable after initiation of the intervention compared with pre-intervention (akin to a difference-in-differences of slopes) (17) (Figure 1). ε_t_ is the random error term representing the unknown variation component of the regression model. The AC-test command was conducted to assess autocorrelation, and the autocorrelation results were both present at lag1 (18). *p* values < 0.05 were considered statistically significant.

Through this model, we aimed to understand whether the DIP reform had a different influence on the health outcomes of UEBMI and URRBMI inpatients. By observing the change of β3, we could interpret the effect of the reform on the number of inpatients, cost, LOS, and ACR for the URRBMI group. And we compared the difference in effects between the two groups via β7. If the reform affected the two groups equally, there would be no significant statistical difference for β7; otherwise, it would be the opposite.

## Results

### Descriptive statistics

The distribution of gender, age groups, and total costs of the two groups were analyzed (Table 1). No statistically significant differences in the distribution of gender (*p* = 0.715 > 0.05) were observed, but a significant difference in age distribution was found between the two groups (*p* value = 0.000). The distribution of total costs manifested a positively skewed distribution (Shapiro–Wilk W test value = 28.469; *p* value = 0.000), with a mean of 12829.69 ± 42.37787, and a median of 8247.29 and the distribution of total costs differed significantly between the two groups (Mann–Whitney test value = −47.596; *p* value = 0.000).

**Table 1:** Description of demographic characteristics.

Variable	Employees	Residents	Test value	*p* value
Gender				
Male	63,600	19,654	0.133	0.715
Female	73,890	22,927		
Age groups				
<18	18,336	10,154	2,718.957	0.000
18–45	18,820	4,761		
>45	10,0334	27,666		
Total costs				
Mean±SD (RMB)	13,545 ± 18,886	10,517 ± 14,553	−30.446	0.000
Median (RMB)	8,672	6,699.5	−47.596	0.000

### Overall analysis for DIP implementation effect

A total of 42,581 URRBMI patients were admitted to the four hospitals between 2019 and 2021. Notably, there was a higher proportion of urban workers (137,490 employees) than residents. In terms of average length of stay (LOS), UEBMI exhibited a longer LOS (11.1 days) than URRBMI (10.3 days). Additionally, the average hospitalization cost for UEBMI stood at 13,545.6 CNY (approximately US$2,099), surpassing that of URRBMI which amounted to 10,517.2 CNY (approximately US$1,630). From a comprehensive perspective on case recovery rates, residents demonstrated an overall cure rate of 68.49%, while urban workers showed a slightly lower rate of 65.71% (Table 2).

**Table 2:** The overall picture of medical service indicators.

Year	Residents				Employees			
	Number of inpatients	Average length of stay (day)	Average of hospitalization costs (RMB)	Actual compensation ratio (%)	Number of inpatients	Average length of stay (day)	Average of hospitalization costs (RMB)	Actual compensation ratio (%)
2019	13,606	10.2	9,769.2	49.93	43,707	11.7	13,283.8	68.76
2020	12,806	10.5	10,433.4	61.82	41,187	11.2	13,492.8	71.99
2021	16,169	10.3	11,213.1	63.40	52,598	10.6	13,804.6	72.12
Total	42,581	10.3	10,517.2	58.62	137,492	11.1	13,545.7	71.01

### Disease spectrum before and after DIP

The number and proportion of patients with diseases of each system were reported. Among them, A-Z excluding P (P stands for certain conditions originating in the perinatal period) represented the infectious and parasitic diseases, tumors, blood and hematopoietic organs, endocrine and other systemic diseases, respectively. Furthermore, no significant difference was found in the types of diseases in discharged patients before and after the implementation of DIP (Table 3).

**Table 3:** The distribution of disease types before-and-after DIP.

The first letter of ICD	Pre-implementation		Post-implementation		*p*
	Number	Percentage (%)	Number	Percentage (%)	
A	594	36.64	1,027	63.36	0.236
B	786	48.46	836	51.54	
C	3,612	46.73	4,118	53.27	
D	1,212	34.41	2,310	65.59	
E	1,410	40.98	2,031	59.02	
F	114	39.18	177	60.82	
G	2,248	41.32	3,193	58.68	
H	1,407	44.77	1,736	55.23	
I	15,614	42.97	20,727	57.03	
J	18,108	53.00	16,057	47.00	
K	10,420	41.59	14,635	58.41	
L	467	43.00	619	57.00	
M	12,112	46.71	13,817	53.29	
N	5,112	41.85	7,103	58.15	
O	116	62.03	71	37.97	
Q	139	41.00	200	59.00	
R	1,767	48.70	1,861	51.30	
S	3,107	43.30	4,068	56.70	
T	1,053	50.12	1,048	49.88	
Z	1,807	35.86	3,232	64.14	
Total	81,205	45.10	98,866	54.90	

### Analysis of DIP implementation effect

**Figure 2:**
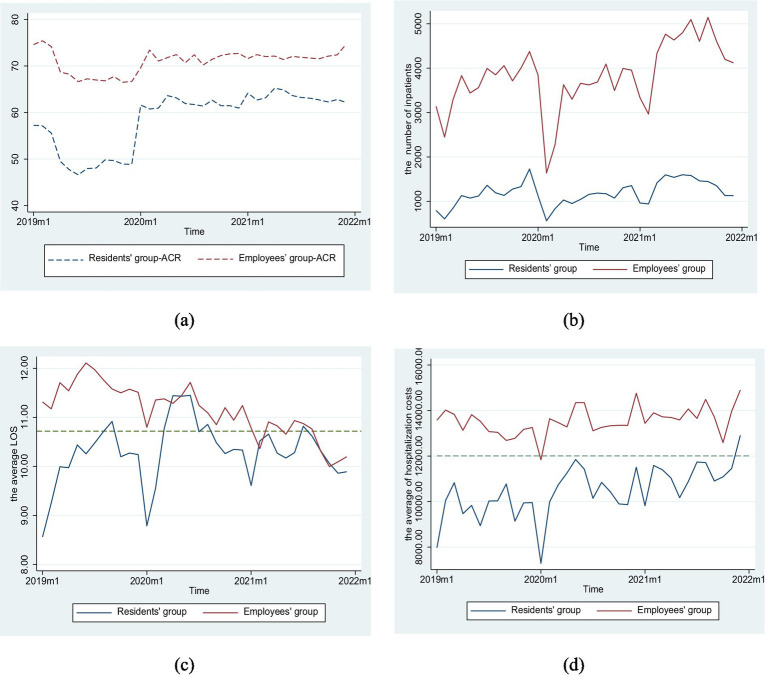
Trend changes regarding efficiency, quality, and burden of inpatients in two groups. **(a)** The trends of actual compensation ratio, **(b)** The trends of number of inpatients, **(c)** The trends of average length of stay, **(d)** The trends of average of hospitalization costs.

In terms of health service efficiency, the number of inpatients in both groups exhibited an upward trend following the implementation of DIP, with increases of 22.26 and 21.59%, respectively. Additionally, the average length of stay (LOS) for URRBMI experienced a minimal variation at only 0.19%, while the LOS for UEBMI decreased by 7.10%. Hospitalization costs for both groups increased by 10.44 and 2.97%, respectively after DIP payment was introduced. Regarding the burden of hospitalization admission, ACR for URRBMI following DIP implementation increased remarkably (15.36%), whereas that for UEBMI increased only slightly (3.07%). Furthermore, throughout this period, the ACR of UEBMI consistently remained higher than that of URRBMI (Table 4; Figure 2).

**Table 4:** The comparison results of various indicators before and after DIP.

Indicators	Residents			Employees		
	Pre-DIP-implementation	Post-DIP-implementation	Rate of change (%)	Pre-DIP-implementation	Post-DIP-implementation	Rate of change (%)
Number of inpatients	19,158	23,423	22.26	62,047	75,443	21.59
Average length of stay	10.32	10.35	0.29	11.55	10.73	−7.10
Average of hospitalization costs	9,945.87	10,984.06	10.44	13,328.6301	13,724.15	2.97
Actual compensation ratio (%)	54.40	62.76	15.36	69.93	72.08	3.07

### The results of multi-group interrupted time series analysis

**Figure 3:**
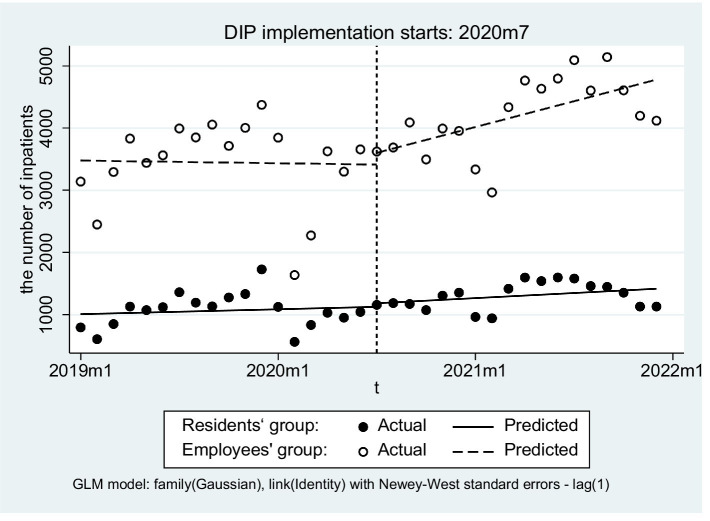
Trends of the number of inpatients before and after DIP reform.

There was no significant difference in the slope of the number of inpatients before and after the implementation of DIP in the URRBMI group (β_3_ = 0.020, *p* = 0.134), the difference in the slopes of the number of inpatients before and after the implementation of DIP between the two groups was also not significant (β_7_ = −0.016, *p* = 0.447) (Figure 3).

**Figure 4:**
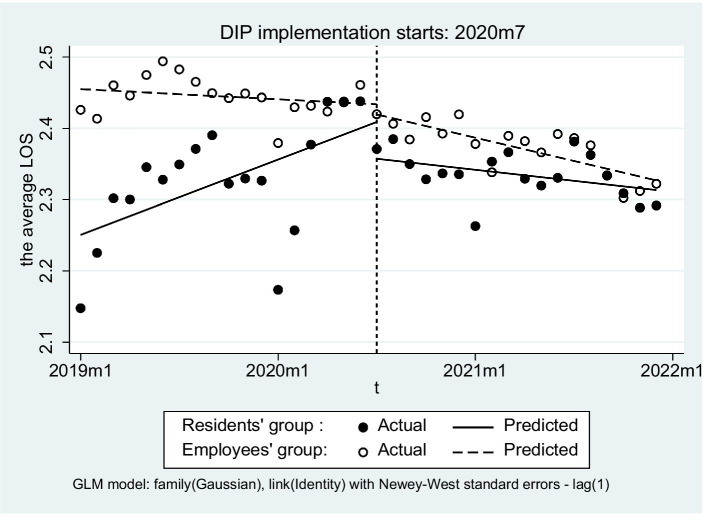
Trend comparison of the average LOS of inpatients before and after DIP.

**Figure 5:**
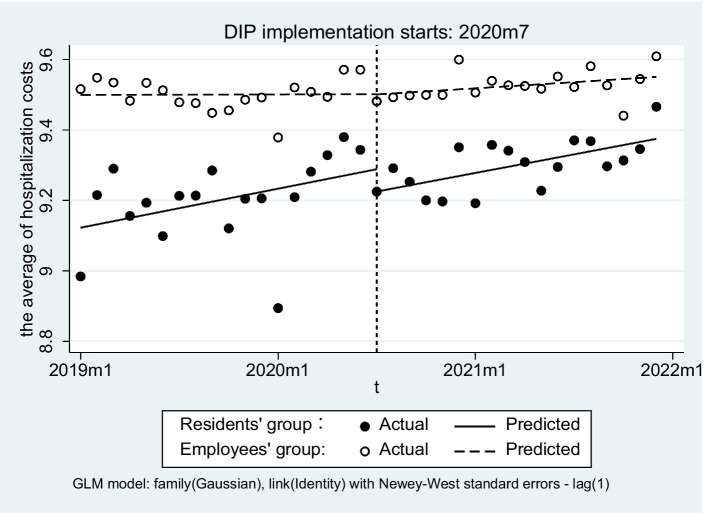
Variation of the average hospitalization costs before and after DIP.

After DIP implementation, URRBMI inpatients showed a significant reduction in the average length of stay (LOS) (β_3_ = −0.004, *p* = 0.014) but no significant change in hospitalization costs on average was observed (β_3_ = 0.003, *p* = 0.382) (Table 5). Between the two groups, however, little differences were found in either the slope of the average LOS (β_7_ = −0.007, *p* = 0.099) or the average of hospitalization costs (β_7_ = −0.003, *p* = 0.630) differed (Figures 4, 5).

**Table 5:** The ITSA results of efficiency, quality, and burden of inpatients.

Variables	Ln (the number of inpatients)		Ln (the average length of stay)		Ln (the average hospitalization costs)		Ln (Actual compensation ratio)	
	Coefficient	*p*	Coefficient	*p*	Coefficient	*p*	Coefficient	*p*
β_0_	8.147	0.000*	2.455	0.000*	9.499	0.000*	4.250	0.000*
β_1_	−0.003	0.796	−0.001	0.401	0.000	0.976	−0.001	0.816
β_2_	0.098	0.556	−0.014	0.347	−0.000	0.985	0.031	0.235
β_3_	0.020	0.134	−0.004	0.014*	0.003	0.382	−0.010	0.617
β_4_	−1.269	0.000*	−0.204	0.000*	−0.377	0.000*	−0.360	0.000*
β_5_	0.009	0.608	0.001	0.012*	0.009	0.135	−0.001	0.984
β_6_	−0.028	0.907	−0.037	0.044*	−0.062	0.429	−0.001	0.984
β_7_	−0.016	0.447	−0.007	0.009*	−0.003	0.630	−0.012	0.087

^*^p < 0.05.

**Figure 6:**
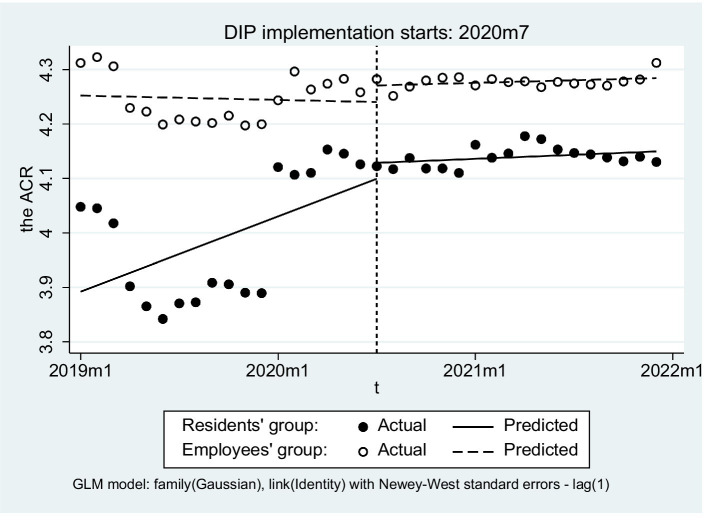
The change of the actual compensation ration of inpatients.

The slope of the ACR did not differ significantly either for URRBMI patients before and after DIP implementation or between the two groups (β_7_ = −0.012, *p* = 0.087) (Figure 6). The only distinction observed in the change of intercept prior to the intervention between the two groups was noted (β_4_ = −0.360, *p* < 0.01).

### Subgroup analysis

To ensure robustness of the findings, the participants were categorized into three age groups: <18 years old, 18–45 years old, and >45 years old. Subsequently, independent subgroup analyses for time series were conducted (Table 6). After DIP implementation, both the slope of average length of stay (LOS) (*p* = 0.022) and average hospitalization costs (*p* = 0.001) differed significantly between the two groups in the under-18 age group (*n* = 28,490). In the 18–45 age group (*n* = 23,580), however, no such differences were observed regarding slopes between employees and residents; only the number of inpatients and average LOS for residents differed significantly in the slope post-DIP implementation (*p* = 0.031 < 0.05). Notably, the over-45 age group (*n* = 128,001) inpatients showed a significant difference in the hospitalization costs slope for both groups. (β_7_ = 0.015, *p* = 0.047 < 0.05).

**Table 6:** The analysis result of the number of inpatients, average length of stay, average of hospitalization costs and actual compensation ratio in subgroups.

Sub-groups	Variable	The number of inpatients	Average length of stay	Average of hospitalization costs	Actual compensation ratio
<18 years old (*N* = 28,490)	β_3_	−0.115 (0.148)	0.001 (0.092)	−0.003 (0.949)	0.001 (0.401)
	β_7_	0.140 (0.086)	−0.051 (0.022*)	−0.012 (0.001*)	−0.004 (0.143)
18-45 years old (*N* = 23,580)	β_3_	0.037 (0.009*)	−0.007 (0.031*)	−0.004 (0.473)	0.001 (0.871)
	β_7_	0.036 (0.198)	0.003 (0.707)	0.003 (0.803)	0.005 (0.364)
>45 years old (*N* = 128,001)	β_3_	0.027 (0.026*)	−0.005 (0.017*)	0.002 (0.594)	−0.001 (0.706)
	β_7_	−0.036 (0.089)	−0.004 (0.360)	0.015 (0.047*)	−0.011 (0.270)

The *p*-value is in parentheses, and * is *p* < 0.05.

## Discussion

In this study, 180,071 cases of inpatients from 4 medical institutions in City C from 2019 to 2021 were used to establish a multiple group ITSA, and to explore the impact of the DIP payment policy on different medical insurance inpatients. The results show that after the implementation of DIP, the average LOS decreased, and the ACR increased for both UEBMI and URRBMI inpatients, indicating positive effects in enhancing services quality and relieving financial burden for inpatients.

As a prospective payment system, DIP effectively incentivizes hospitals to improve efficiency. It encourages hospitals to limit per-case services and treat more patients (19). This is evidenced by the increased number of inpatients in both UEBMI and URRBMI groups after the reform. The significant 7.10% year-on-year decrease in LOS for UEBMI inpatients shows enhanced hospital self-management and diagnostic-treatment efficiency. DIP assigns different point values to diagnosis groups to describe resource consumption (20), and hospitals can earn more points by increasing admissions and treating complex cases, which is called “rush points.” Higher point totals theoretically lead to more revenue. Our study, consistent with previous ones, found that inpatient numbers increased post-DIP reform, with consistent upward trends across populations, indicating DIP’s positive incentives on hospital services in the initial policy implementation stage (21, 22). This aligns with the economic theory of prospective payment, where hospitals are motivated to optimize resource utilization to maximize their revenue within the budget constraints (23).

However, the increase in hospitalization costs for both groups is a concern. While the reduction in LOS implies potential cost-savings, the upward trend in costs indicates that these savings may not be realized. This could be due to several reasons. From the economic theory of provider behavior under prospective payment, the principal-agent theory can be applied to explain this phenomenon. In the context of DIP, hospitals (agents) may face incentives from the payer (principal, i.e., the insurance system). To maximize their utility (such as revenue), hospitals may reduce LOS to treat more patients and earn more points, but at the same time, increase the intensity of services per case. For example, they might order more expensive diagnostic tests or use more costly treatment methods, which leads to an increase in per case costs. Utility maximization frameworks also suggest that providers will make decisions based on a balance between the costs and benefits of different service-providing strategies (24). In this case, the benefits of treating more patients under DIP may outweigh the costs of increasing service intensity per case.

Research shows that, in response to changes in public payments, hospital cost-shifting does exist and is inevitable (25, 26). However, due to the limited availability of cost data in this study, it remains unclear whether there is cost-shifting behavior in hospitals. If hospitals attempt to cut costs in areas such as staff training or medical equipment maintenance to deal with the potential depreciation of points under the regional global budget, this may compromise the quality of medical services.

Regarding patient burden, although the ACR increased for both UEBMI and URRBMI inpatients, the rise in hospitalization costs still needs to be considered. Higher costs may reflect increased service intensity or price inflation rather than genuine value. If the increase is due to price inflation, it may put additional financial pressure on patients, especially those with lower income or under the URRBMI, who may have a relatively lower ability to afford medical expenses.

Thus, this requires policy-making departments to further investigate price setting, provider incentives, and billing practices. Cost audits and quality assurance mechanisms should be an essential part of the DIP implementation. For hospital administrators, the research findings suggest the need for capacity building in cost management, the accuracy of DIP coding, and quality monitoring. It is also crucial to train clinicians to provide efficient care within the DIP constraints. It is advisable that medical institutions explore ways to strengthen discipline construction, guarantee high-quality and reduce patients’ burden.

Meanwhile, different risk pools (UEBMI vs. URRBMI) may respond differently to payment stimuli. Information asymmetry exists between insurance schemes and providers. Providers may have more information about the actual cost and quality of services, which could lead to strategic behavior (27, 28). For example, they may be more likely to admit patients with certain types of diseases or conditions that are more profitable under DIP. Moreover, the administrative capacity of different insurance schemes varies. UEBMI, which mainly covers employed adults, may have a more standardized and efficient administrative process compared to URRBMI, which serves a more diverse population including students, rural residents, and self-employed individuals. This difference in administrative capacity may affect how the DIP policy is implemented and monitored, and ultimately influence the behavior of providers and the outcomes for patients.

### Limitations

This study has several limitations. First, it only considered basic patient-level factors like age and disease categories, ignoring case mix, disease severity, characteristics of hospital grades, clinical practices, etc. This oversight could have introduced confounding variables, biasing the evaluation of the DIP payment policy. Second, it only examined the short-term effects of the DIP policy. Since policy impacts may be delayed, a longer-term study is needed for a more accurate assessment. Third, the research’s scope is limited by hospital selection bias. Conducted on just four hospitals in one city, the findings may not apply to other regions, reducing the study’s generalizability. Fourth, establishing causality is challenging. Without a proper control group or randomized design, residual confounding remains. Even with the MGITSA method, unmeasured factors may still skew the relationship between the DIP policy and outcomes. Finally, the study does not explore how healthcare providers and patients reacted to the DIP policy changes. Understanding these behavioral responses is crucial for fully evaluating the policy’s effectiveness and potential side effects. Future research should include a longer observation period, patient outcome metrics, and qualitative data to better understand provider and patient responses. Comparative studies across regions and payment models would also enhance the policy discussion.

## Conclusion

In conclusion, although DIP demonstrates positive effects on hospital efficiency and patient reimbursement coverage in the short term, issues such as the increase in hospitalization costs, the lack of exploration in ethical and behavioral economic aspects, potential differential impacts, and concerns over rising costs require further research. Future studies should focus on these areas, aiming to offer more comprehensive insights for healthcare providers and policymakers. Meanwhile, continuous monitoring and policy adjustment are needed to ensure the effectiveness and sustainability of DIP, given its role as an effective tool for improving hospital efficiency and reducing patients’ economic burden.

The Chinese government has been advancing the reform of DIP payment methods for medical insurance nationwide. It is imperative to conduct a comprehensive analysis of the differential impacts of payment reforms on various categories of medical insurance globally, which will provide valuable insights for healthcare providers and policymakers seeking to regulate medical practices effectively (19).

## Data Availability

The data analyzed in this study is subject to the following licenses/restrictions: the health data was anonymously provided by 4 hospitals in City C, it is not suitable for public disclosure. Requests to access these datasets should be directed to Lixiang Wu, 871618885@qq.com.
